# Real-time Utilization of Metagenomic Sequencing in the Diagnosis and Treatment Monitoring of an Invasive Adenovirus B55 Infection and Subsequent Herpes Simplex Virus Encephalitis in an Immunocompetent Young Adult

**DOI:** 10.1093/ofid/ofy114

**Published:** 2018-05-16

**Authors:** Xueling Fang, Mi Xu, Qiang Fang, Haiqin Tan, Jiale Zhou, Ziqin Li, Fan Li, Shangxin Yang

**Affiliations:** 1 Department of Intensive Care Unit, The First Affiliated Hospital, College of Medicine, Zhejiang University, Hangzhou, China; 2 IngeniGen XunMinKang Biotechnology Inc., Hangzhou, China; 3 Zhejiang-California International Nanosystems Institute, Zhejiang University, Hangzhou, China; 4 Department of Pediatrics, Mattel Children’s Hospital, University of California Los Angeles, Los Angeles, California; 5 Department of Pathology, University of New Mexico, Albuquerque, New Mexico

**Keywords:** adenovirus B55, fever of unknown origin, HAdV-B55, HSV-1 Encephalitis, ICU, pneumonia, ribavirin, shotgun metagenomics, viremia

## Abstract

**Background:**

Metagenomic sequencing has shown tremendous promise in solving difficult infectious diseases cases. In this study, we utilized this technology to help guide the care of a critically ill patient with severe pneumonia, fever of unknown origin, and subsequent encephalitis in the intensive care unit (ICU).

**Methods:**

Shotgun metagenomic sequencing was performed on the patient’s blood, bronchoalveolar lavage (BAL), and cerebral spinal fluid by using an Illumina MiniSeq sequencer.

**Results.:**

A high load of human adenovirus B55 (HAdV-B55), a highly pathogenic adenovirus associated with numerous recently reported outbreaks and deaths in China, was detected in both blood and BAL, which explained the severity of the condition. The patient was treated with intravenous ribavirin, which cleared the virus after 26 days. Metagenomic sequencing also helped diagnose an unexpected herpes simplex virus–1 encephalitis during hospitalization, which led to timely treatment.

**Conclusions.:**

This was the first successful case utilizing metagenomic sequencing to guide diagnosis and treatment in the ICU setting in China. We have proven the concept that metagenomic sequencing can play an important role in determining clinical approaches and ultimately in improving patient outcomes. We also hope to share our successful treatment protocol for the severe pneumonia and viremia caused by HAdV-B55.

## INTRODUCTION

Next-generation sequencing (NGS)–based shotgun metagenomics, a method that directly sequences all the DNA in a sample at once, has shown tremendous potential for resolving difficult-to-diagnose infections due to its high sensitivity and nearly unlimited coverage of pathogen detection compared with conventional culture-dependent clinical microbiology and nucleic acid amplification tests [1, 2]. However, most published successful cases utilizing metagenomics for diagnosis are limited to meningoencephalitis, as there is much less human DNA in the cerebral spinal fluid (CSF) that can dilute out pathogen DNA and the sterility of the CSF reduces the potential for other microbial contaminants. In the intensive care unit (ICU), accurate diagnosis of many other critical conditions including severe pneumonia, sepsis, and fever of unknown origin is also very challenging and represents even greater unmet clinical need. In this study, we used shotgun metagenomics for pathogen detection in various specimen types including bronchoalveolar lavage (BAL), blood, and CSF on 1 critically ill ICU patient in real time to guide diagnosis and treatment throughout the hospitalization course. The metagenomics results provided critical information to rule out other infections and thereby focused on the most important pathogens, which ultimately contributed to the survival and recovery of the patient. Our case provides a proof of concept for precision medicine empowered by real-time pathogen metagenomics testing in the ICU setting.

## CASE REPORT

A 25-year-old previously healthy male patient from Yunnan province in Southern China was airlifted to the First Affiliated Hospital of Zhejiang University for “fever of unknown origin” and respiratory failure on October 29, 2017. Ten days before, he started having a fever of 38°C and mild diarrhea without an obvious etiology. A few days later, he started feeling chest tightness and shortness of breath and having cough with yellow purulent sputum. He was admitted to a local hospital, where a thoracic computed tomography (CT) scan revealed pneumonia with a small amount of pleural effusion in the right lung. He was diagnosed with “lobar pneumonia” and treated with moxifloxacin plus cefoperazone sulbactam for 5 days. However, the symptoms worsened, and the patient continued having a high fever (40°C). Another CT scan showed significant progress of consolidation in the right lung and multiple nodules and pleural effusion in the left lung. The treatment regimen was changed to imipenem, linezolid, caspofungin, and ganciclovir. Methylprednisolone was given as well. However, the patient’s condition rapidly deteriorated to respiratory failure, which required mechanical ventilation, thoracic drainage, and drug sedation, before he was transferred to our hospital. No laboratory results were available from the outside hospital. Personal history revealed the patient to be a heavy smoker.

Upon admission, he was febrile (38.4°C), tachycardic (109 bpm), and hypotensive (62/51 mmHg) with leukocytosis (white blood cell count [WBC] 18.6 X10E9/L). His C-reactive protein (CRP) was 146.42 ng/L, but procalcitonin (PCT) was only 0.49 ng/L. His troponin (0.3 ng/mL) level and his liver enzyme (aspartate aminotransferase [AST] 140 U/L) were both elevated. Bronchoscopy showed profound congestion and edema in the airway mucosa accompanied by multiple bleeding ulcers. Microbiology work-ups were initiated, and the patient received daptomycin (for suspected endocarditis after bedside echocardiogram showed hyperechoic response in the right ventricle) and piperacillin/tazobactam. On hospital day (HD) 2, the respiratory viral polymerase chain reaction (PCR) panel detected adenovirus in the sputum. Ganciclovir (0.3 g qd) was started again, and immunoglobulin was added. However, the exact origin of the fever was unclear, and the severity of the condition didn’t seem to match adenovirus respiratory infection alone. A transesophageal echocardiography (negative results) was performed to rule out infective endocarditis. Bone marrow puncture and lymph node ultrasonography (negative results) were performed to rule out lymphoma. These revealed T-cell subpopulations in the bone marrow suggestive of suppressed immune status, and therefore thymosins was given. On HD 3, BAL and blood were sent for shotgun metagenomics testing to identify the source of the fever. On HD 4, BAL and sputum culture came back positive with *Aspergillus fumigatus*; thus voriconazole was started. At this point, the diagnosis of the unknown fever was starting to shift to a fungal pneumonia. On HD 5, the shotgun metagenomics results came back positive with large amount of adenovirus in both BAL (1301 reads) and blood (795 reads). No other pathogens, including bacteria, fungus, and parasites, were found. *Aspergillus fumigatus*, which was isolated in the initial sputum culture, was not detected by the shotgun metagenomics. Because the subsequent respiratory cultures also did not yield any fungus, combined with the shotgun metagenomics results, the 1-time finding of *A. fumigatus* in the sputum was deemed to be a result of environment contamination. The high number of adenovirus reads were sufficient for de novo assembly into several large contigs (max size, 4800 bp), which were closely matched to human adenovirus B55 (HAdV-B55). Of note, this is the same strain that has been reported to cause numerous outbreaks in military units, hospitals, and schools in China, and that often causes deaths in immunocompetent young patients [3–7]. With this information, it was very clear that this patient was suffering an invasive HAdV-B55 infection that started from pneumonia and progressed into viremia. All clinical efforts were refocused to antiviral therapy. Because cidofovir, the firstline drug for adenovirus infection, is not available in China, ganciclovir was replaced by ribavirin (0.5 g q12h) administered intravenously on the basis of more successful treatment cases reported on the literature [8–10].

On HD 8, the patient’s inflammatory markers, including WBC, CRP, and PCT, all increased significantly. Bronchoscopy showed an increased amount of purulent sputum, and the culture grew pan-drug-resistant (only susceptible to tigecycline) *Acinetobacter baumannii*, a common nosocomial pathogen circulating in many ICUs in China, including ours [11]. Piperacillin/tazobactam was then replaced by cefoperazone/sulbactam (1 g q6h) plus tigecycline (50 mg q12h). To rule out other infections and monitor the antiviral therapy, a second set of BAL and blood samples was sent for shotgun metagenomics on HD 9. The results were negative in the blood but positive in the BAL, with a reduced load of adenovirus (10 reads) and a high load of *A. baumannii* (1666 reads). No other pathogens were detected in the BAL. The absence of adenovirus in the blood (confirmed by PCR) and the significant decrease of adenovirus load (1 log reduction by a quantitative PCR) in the BAL suggested that the patient was responding to the ribavirin treatment. The detection of *A. baumannii* was consistent with the culture results. The absence of any fungus in the BAL by metagenomics testing again, supported by repetitive negative fungal culture in the respiratory samples, prompted the discontinuation of voriconazole. Ribavirin treatment was continued, and a qualitative adenovirus PCR test on the sputum was done daily to monitor viral infections in the lung. On HD 10, the patient’s liver enzymes, troponin level, and ejection fraction had all returned to their normal ranges. On HD 18, due to repeated positive cultures of *A. baumannii* in the sputum, the dose of cefoperazone/sulbactam was increased from 1 g q6h to 3 g q6h. On HD 21, the adenovirus PCR in the sputum finally turned negative, the amount of *A. baumannii* grown in sputum culture also turned from heavy to rare, and the inflammatory markers (WBC/CRP/PCT) had all dropped significantly. On HD 24, bronchoscopy showed significant improvement in the trachea and the airways; thus, the chest drainage tube was removed, and the antibiotics were discontinued. On HD 26, the patient woke up after sedation was discontinued. On HD 28, due to the concern for drug-induced liver damage (AST increased to 161 U/L), ribavirin was discontinued. His condition began to stabilize, and ventilator support was gradually reduced.

**Figure 1.**
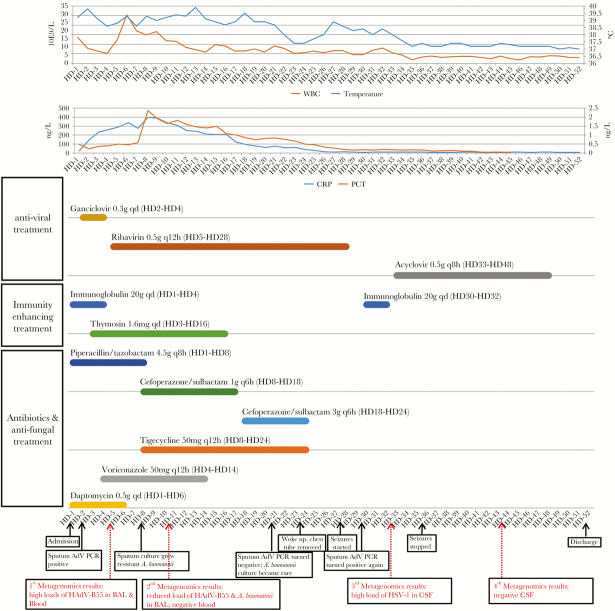
Summary of inflammatory markers, treatment, major events, laboratory findings, and metagenomics results during the whole hospitalization course. Abbreviations: BAL, bronchoalveolar lavage; CRP, C-reactive protein; CSF, cerebrospinal fluid; PCR, polymerase chain reaction; PCT, procalcitonin; WBC, white blood cell.

However, on HD 28, the patient developed altered mental status, seizures, and muscle weakness. Head CT showed widening in the sulci and cisterns, concerning for encephalitis. Meanwhile, the patient started having fever again (38.6°C). On HD 30, the sputum adenovirus PCR test was positive again, raising concern for neurological adenovirus infection. A lumbar puncture was performed and the CSF, which surprisingly had unremarkable biochemistry (glucose 4.3 mmol/L, protein 0.331 g/L) and cellular (RBC 170/uL, WBC 33/uL, 12% neutrophils, 88% lymphocytes) results, was sent for shotgun metagenomics testing. Two days later, the metagenomics results came back positive for herpes simplex virus–1 (HSV-1; 844 reads), which was subsequently confirmed by HSV-1 PCR. Neither adenovirus nor any other pathogens was detected. A diagnosis of HSV-1 encephalitis was made, and high-dose acyclovir (500 mg Q8h) was given to the patient. After 3 days of acyclovir treatment, the fever and seizures were gone, and the limb muscle strength was improved. On HD 40, the patient showed substantial improvement and could eat unassisted; therefore, the nasogastric intubation was removed. On HD 44, ventilation support was finally withdrawn. Another lumbar puncture was done, and the CSF was sent for shotgun metagenomics testing, which came back negative (confirmed by HSV-1 PCR). The patient was discharged on HD 52. Blood cultures were never positive, and the patient’s adenovirus PCR in the sputum was intermittently positive until HD 48. The hospitalization history, including major events, treatment courses, inflammatory markers, and test results, is summarized and illustrated in Figure 1.

## METHODS

### Setting and Ethical Approval

This study was approved by Ethics Committee at the The First Affiliated Hospital of Zhejiang University under the clinical research project titled “Improving Treatment for Severe Pneumonia by Using Emerging Diagnostic Technologies and Therapeutics in the ICU Setting” (project #2017ZX10204401003004). All participants including the patient described in this paper were informed about the study, and written informed consents were obtained.

### Shotgun Metagenomic Sequencing

Low-speed centrifugation (1500 g for 20 minutes) was performed to remove human cells in the samples including BAL, blood, and CSF. For the blood, only the plasma was collected for further testing. Samples were then homogenized using bead beating followed by DNA extraction using the IngeniGen Total Nucleic Acids Extraction Kit (IngeniGen Biotechnology, Hangzhou, China). DNA libraries were prepared using the IngeniGen DNA Library Prep Kit following the manufacturer’s protocols. Briefly, the DNA was fragmented, and the Illumina-compatible adaptors were added to the fragmented DNA simultaneously by a tagment enzyme. The library was purified by magnetic beads and then amplified by 15 PCR cycles. Sequencing was performed on the Illumina MiniSeq (Illumina, San Diego, CA) using 2X150bp chemistry. A negative control was included in each run to detect the background contaminants, and an internal control (a unique marine bacteria suspension, 100 CFU/mL) was added to each sample to monitor the entire process. Data analysis was performed using IngeniSeq MG (IngeniGen Biotechnology, Hangzhou, China), a proprietary automated shotgun metagenomics analysis platform for pathogen detection. Briefly, human and other contaminant sequences that were known to be derived from the reagents were removed from the raw data, and the filtered sequences were de-duplicated and then matched against a curated database consisting of more than 10 000 microbial reference genomes. The resulting hits were again filtered by a proprietary algorithm that further removed background contaminants that may appear during sample processing and the library preparation, resulting in a final report of detected pathogens. The quality control matrix is briefly described here: (1) a true-positive result is called only when the negative control has corresponding reads <10% compared with the sample; (2) the internal control should have reads >50 for the results to be valid in each sample.

### Adenovirus Genetic Analysis

Sequence reads aligned to adenovirus were de novo assembled into contigs using Geneious (Biomatters, New Zealand). The 10 longest contigs were matched to the most closely related adenovirus genomes using BLAST. The adenovirus reads were then mapped to the appropriate reference genome, and the mapping statistics (eg, percent identical sites, percent pairwise identity, and percent coverage [Ref-Seq%]) were calculated by Geneious.

### Quantitative Adenovirus and HSV-1 PCR

The quantitative PCR assays for adenovirus and HSV-1 were performed using the IngeniGen Adenovirus PCR Kit and IngeniGen HSV-1 PCR Kit (IngeniGen Biotechnology, Hangzhou, China) on the same DNA used for shotgun metagenomics. The real-time PCR analyses were run on an ABI 7500 (ThermoFisher Scientific, Waltham, MA), and quantification was based on standard curves generated by running 10-fold serial dilutions of quantified plasmids carrying the target sequences.

## RESULTS

**Figure 2.**
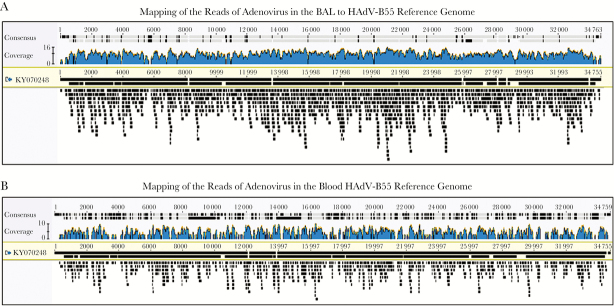
Mapping results of adenovirus reads in the bronchoalveolar lavage (BAL) and blood to a closely related HAdV-B55 reference genome. A, Mapping results of adenovirus reads in the BAL to HAdV-B55 reference genome KY070248. B, Mapping results of adenovirus reads in the blood to HAdV-B55 reference genome KY070248.

Between 2 million and 5.8 million sequencing reads were acquired for each sample, with Q30 percentages all >90.0%. The pathogens detected with their corresponding read counts are shown in Table 1. Quantitative PCR tests for adenovirus and HSV-1 were performed on the same DNA to confirm the presence of the viruses detected and quantify the viral load. After 6 days of intravenous ribavirin treatment, the metagenomics test showed that the adenovirus in the BAL had decreased from 1301 sequence reads to only 10 sequence reads. The significant reduction of adenovirus load was confirmed by quantitative PCR (from 2 814 889 copies/mL to 211 743 copies/mL, 1 log difference). In the blood, a more dramatic reduction of the adenovirus load was found by both metagenomics (from 795 reads to 0 reads) and quantitative PCR (from 21 655 copies/mL to undetectable) after treatment. Adenovirus sequence reads were assembled using the Geneious assembler with default settings. In the BAL sample, 1279/1301 reads were used to produce 5 contigs with a total length of 34 738 bases (N50, 2007 bp). In the blood sample, 743/795 reads were used to produce 125 contigs with a total length of 23951 bases (N50, 213 bp). The most closely related adenovirus reference genome was found to be human adenovirus 55 isolate 60-GD-2016 (accession KY070248; genome size, 34 759 bp), which was isolated from an outbreak in a neurosurgical department of a general hospital in Guangdong, a province in southern China during the summer of 2016 [3]. Genetic analysis by mapping the adenovirus reads from the BAL to this genome showed 97.7% coverage with 98.2% pairwise identity and 94.8% identical sites. Similarly, the mapping of the reads from the blood showed 76.1% coverage with 98.3% identity and 99.0% identical sites (Figure 2). The metagenome data with human DNA sequences removed were submitted to NCBI (BioSample accessions SAMN08939555, SAMN08939556).

**Table 1.** Result Summary of Shotgun Metagenomics and Quantitative Real-time PCR on the Patient Samples

Sample (Hospital Day)	Shotgun Metagenomics Results					Quantitative Real-time PCR Results
	Total NGS Reads	Reads After Host DNA Removal	Reads Aligned to Bacterial Database	Reads Aligned to Nonbacterial Database	Pathogens Detected After Filtering, Reads	Viral Load, Copies/mL
BAL (HD3)	5 834 434	95 300	484	2865	Adenovirus (1301)	2 814 889
BAL (HD9)	2 028 030	43 139	2483	367	Adenovirus (10), *Acinetobacter baumannii* (1666)	211 743
Blood (HD3)	4 867 325	125 375	4648	2450	Adenovirus (795)	21 655
Blood (HD9)	4 076 330	114 449	7774	337	Negative (0)	Not detected
CSF (HD30)	3 492 920	107 793	1985	1302	HSV-1 (844)	20580
CSF (HD44)	2 934 620	80 613	4398	328	Negative (0)	Not detected

Abbreviations: BAL, bronchoalveolar lavage; CSF, cerebrospinal fluid; NGS, next-generation sequencing; HSF-1, herpes simplex virus–1; PCR, polymerase chain reaction.

## DISCUSSION

An HAdV-B55-associated outbreak was first reported in a senior high school with 254 patients infected in Shaanxi, China, in 2006 [4]. Since then, numerous outbreaks have been reported from northern China (Shaanxi, Hebei, Henan, Shangdong, Beijing, Tianjin, Chongqing, and Gansu) [5–7, 12–14], and recently more from the southern and western regions (Tibet, Sichuan, Yunnan, Guangdong) [3, 15]. Notably, our patient was from Yunnan. HAdV-B55 outbreaks have typically happened in crowded areas, including military bases, hospitals, schools, and physical training facilities. Genomic analysis showed that HAdV-B55 evolved from a recombination between HAdV-B14 and -B11 [16]. In vitro studies showed that HAdV-B55 exhibited higher levels of replication in respiratory cells than did either of its parents [17]. Worldwide, although HAdV-B14 and -B11 have caused many outbreaks in Asia, Europe, and the United States, especially among military populations [18–20], HAdV-B55 outbreaks were almost exclusively reported in China. Clinically, HAdV-B55 seems to be highly pathogenic, with numerous fatal cases reported in immunocompetent young patients [6, 7, 13]. Most fatal cases have been reported in males, but no clear risk factors have been identified. Only 1 fatal case report described the patient as a heavy smoker [6]. Of note, our patient is also a heavy smoker. In most severe and fatal cases, patients start with a sudden onset of respiratory distress that progresses rapidly to respiratory failure, often with imaging showing “lobar pneumonia.” Many patients infected with HAdV-B55, including ours, also had viremia [6, 7, 13]. In 1 study, higher initial HAdV-B55 viral load in the respiratory tract and sustained viremia (more than 2 weeks) were found to be associated with poor outcomes [7]. Therefore, lowering the HAdV-B55 viral load, as demonstrated by the literature and our case, seems to be the most effective strategy to optimize patient outcomes.

For treatment, because cidofovir, the firstline drug for adenovirus infection, is not currently available in China, acyclovir, ganciclovir, and ribavirin have all been utilized to treat HAdV-B55 infection [7, 13], although the efficacy of each antiviral drug could not be assessed due to the limited number of patients and lack of a controlled study [8, 9]. In our case, we treated the patient with ribavirin combined with immunoglobulin (4 days) and then thymosin (14 days). The patient responded with a significant viral load reduction in the respiratory tract (>1 log) and resolving of viremia within the first 6 days of treatment. The adenovirus in the respiratory tract was eventually cleared 20 days after the completion of a total of 24 days of intravenous ribavirin treatment.

Interestingly, drug-resistant *A. baumannii* nosocomial infection has often been reported in severe HAdV-B55 infection–related cases in China [6, 13], resulting in at least 1 reported death directly related to the bloodstream infection by this bacterium [13]. In our case, the patient also developed ventilation-associated nosocomial infection by a pan-drug-resistant *A. baumannii* and had to be treated with combination antibiotic therapy for a prolonged time (17 days). The high prevalence of nosocomial infections caused by this “superbug” highlights the urgency and challenges in ICUs in China, where much tighter infection prevention policies and enforcement are needed [11].

As adenovirus can cause encephalitis [21], when our patient first presented with seizures and altered mental status, especially right after the discontinuation of the ribavirin treatment, the suspicion for adenovirus encephalitis was very high. Surprisingly, the metagenomics results showed that the infectious etiology was actually HSV-1. Although HSV-1 is a frequent cause of encephalitis, especially in immunocompromised patients [22], in-hospital HSV-1 encephalitis is very rare except for patients with certain known risk factors such as neurological surgeries or the use of anti-inflammatory drugs [23]. Our patient was immunocompetent, did not have any recent surgery, and was not given anti-inflammatory treatment. Therefore, HSV-1 encephalitis after 24 days of hospitalization was unexpected, and the source (primary vs reactivation) of the infection remain unclear. Fortunately, the patient responded very quickly to acyclovir treatment and recovered completely.

The highlight of this case report is the utilization of shotgun metagenomics testing for pathogen detection, monitoring, and rule-out in multiple specimen types in real time throughout the entirety of ICU hospitalization. The metagenomic findings of high adenovirus load in both BAL and blood, as well as the absence of *A. fumigatus,* helped guide the diagnosis of “fever of unknown origin.” Genotyping of the adenovirus as HAdV-B55, a highly pathogenic adenovirus circulating in China, helped explain the severity of the infection and focus clinical efforts on antiviral treatment. The second set of metagenomics findings on BAL and blood samples after 6 days of treatment helped monitor the efficacy of treatment and ruled out pathogens other than *A. baumannii* in the respiratory tract that were causing elevated inflammatory markers. The third set of metagenomics findings on the CSF helped identify HSV-1 as the etiology of encephalitis, which was very unexpected given the prior HAdV-B55 infection. Importantly, the metagenomics testing was instrumental in guiding the correct diagnosis of HSV-1 encephalitis in a timely manner.

This study has several limitations. First, we did not perform RNA-based metagenomic sequencing to rule out RNA viruses. However, in this case, the patient had already tested negative for most respiratory RNA viruses by a respiratory virus PCR panel, and the likelihood of having RNA virus–associated encephalitis (such as enterovirus) after 26 days of hospitalization was low due to a lack of risk factors. In other circumstances, however, both DNA- and RNA-based metagenomics should be performed for a complete pathogen scrutinization. Second, the shotgun metagenomic sequencing in this study was not deep enough (up to 5.8 million reads) to provide sufficient sequences to cover the whole HAdV genome, which prohibited us from performing additional phylogenetic analyses to provide further epidemiological information. Lastly, this is a case report of a single patient. We do not know the prevalence of HAdV-B55 infections among the population in Yunnan province. Further larger-scale molecular epidemiological studies are needed to address this question.

In summary, to our knowledge, this is the first successful case utilizing shotgun metagenomics in real time to help guide the diagnosis and treatment of a critically ill patient in the ICU in China. Metagenomics testing was integrated into the entire course of hospitalization. We have demonstrated the concept that metagenomics testing can play an important role in determining clinical approaches and ultimately in improving patient outcomes. We also hope to share our successful treatment protocol for severe pneumonia and invasive infections caused by HAdV-B55. Given the endemic nature of these pathogens in China, we expect that this case report and literature review could potentially provide guidance to improve the clinical management of and patient outcomes associated with HAdV-B55 infections.
